# Sex differences in the correlation between lipids related to cardiovascular risk factors and small dense LDL particles in patients with type 2 diabetes

**DOI:** 10.20945/2359-4292-2024-0069

**Published:** 2024-10-01

**Authors:** Alena Viktorinova, Robert Brnka, Margita Pirosova, Peter Pontuch, Sona Kinova

**Affiliations:** 1 Institute of Medical Chemistry, Biochemistry and Clinical Biochemistry Faculty of Medicine Comenius University in Bratislava Bratislava Slovakia Institute of Medical Chemistry, Biochemistry and Clinical Biochemistry, Faculty of Medicine, Comenius University in Bratislava, Bratislava, Slovakia; 2 Faculty of Medicine Comenius University in Bratislava University Hospital Bratislava Slovakia 1st Department of Internal Medicine, Faculty of Medicine, Comenius University in Bratislava and University Hospital, Bratislava, Slovakia; 3 Faculty of Medicine Comenius University in Bratislava University Hospital Bratislava Slovakia 4th Department of Internal Medicine, Faculty of Medicine, Comenius University in Bratislava and University Hospital, Bratislava, Slovakia

**Keywords:** Atherosclerosis, cardiovascular risk factors, lipid hydroperoxides, lipid risk markers, small dense LDL particles

## Abstract

**Objective:**

Sex differences in lipid metabolism associated with prevalent small dense (S-) low-density lipoprotein (LDL) cholesterol particles are not elucidated. An LDL to apolipoprotein B (ApoB) ratio < 1.2 can estimate how prevalent S-LDL particles are and, thus, reflect cardiovascular risk. The aim of this study was to evaluate the sex distribution of LDL/ApoB ratio among patients with type 2 diabetes (DM) and to assess, in both sexes, the correlations between key lipid parameters and LDL/ApoB < 1.2.

**Subjects and methods:**

The study included 190 Caucasian participants (mean age 51.8 ± 6.4 years) with DM (DM group) or without DM (control group) divided into subgroups according to sex. The participants were examined for levels of several lipid parameters, selected lipid-related oxidative stress markers, and estimated S-LDL prevalence.

**Results:**

An LDL/ApoB < 1.2 (p < 0.05) was observed in 67% of male and female patients with DM. Although triglyceride levels did not differ between men and women, women had higher levels of total cholesterol (p < 0.05) and LDL cholesterol (p < 0.01) than men. Among women with LDL/ApoB < 1.2, strong correlations were observed between values of lipid hydroperoxides (LOOH) and atherogenic index of plasma (p < 0.005) and between levels of triglycerides and LOOH (p < 0.005) and ApoB (p < 0.0001).

**Conclusions:**

The findings indicate that women with LDL/ApoB < 1.2 tend to have a higher cardiovascular risk than men. Additionally, LDL/ApoB < 1.2 can be a surrogate marker for estimating the S-LDL prevalence in individuals with potentially increased cardiovascular risk.

## INTRODUCTION

The incidence of atherosclerosis-related cardiovascular disease (CVD) varies between men and women worldwide. However, the etiopathogenetic factors involved in these differences are still unclear (1). Notably, CVD usually develops in women several years later than in men and increases dramatically during menopause (2,3). Recent recommendations for the primary prevention of CVD in women have focused on several risk factors associated with changes in sex hormone levels throughout a lifetime (4,5), such as hypertension, dyslipidemia, diabetes mellitus (DM), perimenopause, and others (6,7). Some European specialists have pointed out the lower participation of women in clinical trials and the increased need for prevention, diagnosis, and treatment of CVD in this population (8,9). Clinical studies examining age- and sex-related differences in lipid profile have reported a higher cardiovascular risk in menopausal women compared with men (10-12).

The plasma atherogenic lipoprotein profile is a known important risk factor for atherosclerosis-related CVD (13). The atherogenic index of plasma (AIP) has been proposed as a marker of plasma atherogenicity based on its strong and positive association with cholesterol esterification rates, lipoprotein particle size, and remnant lipoproteinemia (14,15). The retention of low-density lipoprotein cholesterol (LDL-c) and other cholesterol-rich, apolipoprotein B (ApoB)-containing lipoproteins contributes to the development of atherosclerosis (16,17). Recent evidence has pointed to some markers that may estimate how prevalent small dense LDL-c (S-LDL) particles are in an individual, which in turn correspond to values of LDL/ApoB < 1.2 and cardiovascular risk (18,19). The S-LDL particles have a lower affinity for the LDL receptor, a higher cholesterol content, and a longer half-life; they penetrate the arterial wall more easily and are more susceptible to AIP oxidation than larger LDL particles (20). These S-LDL particles have strong atherogenic potential and represent a major risk factor for the development and progression of atherosclerotic CVD (21,22). Given the causal role of LDL-c and ApoB in the pathogenesis of this process, evaluating the prevalence of S-LDL particles appears to be an appropriate method for cardiovascular risk stratification.

Laboratory methods for determining the size of LDL particles are difficult to use in routine clinical practice due to the complexity of their procedures and high cost. On the other hand, the LDL/ApoB ratio (which reflects LDL particle size) can be easily calculated from laboratory-measured LDL-c and ApoB levels. Some researchers have suggested that smaller LDL particle sizes are related to severe lipid abnormalities and potential CV risk (18,19). They have found that the LDL/ApoB value not only correlates positively with LDL particle diameter but also that an LDL/ApoB ratio of 1.2 (or less) reflects an LDL particle size of 25.5 nm (or smaller), indicating the prevalence of S-LDL particles.

Based on this hypothesis, our study aimed to evaluate the sex-related distribution of the LDL/ApoB ratio and estimate the prevalence of S-LDL particles using an LDL/ApoB < 1.2 in patients with DM. Another aim of the study was to investigate differences in the relationships between atherosclerosis-related lipid parameters in men and women with LDL/ApoB < 1.2, which could lead to a better stratification of cardiovascular risk. The clinical interest of this study was focused on highlighting the LDL/ApoB ratio as a surrogate marker in the assessment of cardiovascular risk.

## SUBJECTS AND METHODS

### STUDY POPULATION

The study comprised 190 Caucasian participants divided as follows: 121 men with a mean age of 52.0 ± 6.6 years (83 outpatients with DM [DM-M] and 38 controls [CG-M]) and 69 women with a mean age of 51.5 ± 6.1 years (46 outpatients with DM [DM-F] and 23 controls [CG-F]). The inclusion criteria for patients with DM – who attended the outpatient clinics of the Department of Internal Medicine, Faculty of Medicine, Comenius University in Bratislava – were as follows: men and women aged < 60 years, with previously diagnosed type 2 DM, glucose-lowering therapy, and with/without a history of CVD. Patients with DM received insulin, oral hypoglycemic agents (OHA), a combination of insulin and OHA, a special diet, and lipid-modifying agents. The glycemic control of patients with DM was performed using glycated hemoglobin (HbA1c) levels at the time of enrollment. There were no inclusion or exclusion criteria related to HbA1c. The exclusion criteria were as follows: history of severe hepatic or renal dysfunction, acute coronary or cerebrovascular events, acute infections, and neurological or malignant diseases.

The inclusion criteria for healthy controls were as follows: men and women age-matched to the participants in the DM group and without history of hyperglycemia, DM, hypertension, CVD, lipid abnormalities, or acute infections. All participants were enrolled in the study at the time of their medical examination. In addition, the height and weight of all participants were recorded, and their body mass index was calculated.

### Ethics

This study was conducted in line with the ethical principles for medical research involving human subjects of the World Medical Association Declaration of Helsinki (as revised in 2013). The protocol of the study was approved by the Ethics Committee of the Faculty of Medicine, Comenius University, and the University Hospital, Bratislava (Decision no. 41/2022). Informed consent was obtained from all study participants. The anonymity of the patients was maintained within the manuscript according to ethical principles.

### Biochemical measurements

At the time of enrollment, fasting blood samples were obtained from all participants. The serum was separated by centrifugation under standard conditions, aliquoted into polystyrene tubes (Eppendorf AG, Hamburg, Germany), frozen at -80 °C, and stored until further analysis. Serum levels of triglycerides (TG), total cholesterol (TC), high-density lipoprotein cholesterol (HDL-c), LDL-c, ApoA1, ApoB, and glucose were determined by standardized methods using an automated analyzer (ADVIA 2400 Chemistry System, Siemens, USA) and kits (Erba Lachema, Brno, Czech Republic). The results are expressed as mmol/L or g/L. The HbA1c level in whole blood was measured using the Variant II Turbo Hemoglobin Testing System (Bio-Rad Laboratories, Hercules, CA, USA). Levels of HbA1c are expressed in both traditional Diabetes Control and Complications Trial (DCCT)-derived units (in %) and International Federation of Clinical Chemistry and Laboratory Medicine (IFCC)-recommended SI units (in mmol/mol) (23). All measurements were performed in an accredited laboratory of clinical biochemistry in Bratislava.

Serum levels of lipid hydroperoxides (LOOH) were determined according to a previously described method (24) using a UV-1800 Spectrophotometer (Shimadzu Corporation, Japan) at 365 nm and 37 °C. The molar extinction coefficient for I3 was 24,600 mol-1·dm3·cm-1. The results are presented in nmol/mL of serum.

### Calculation of lipid risk indexes

Lipid risk indexes were calculated from basic lipid parameters according to current guidelines (16). Non-HDL-c was expressed as the difference between TC and HDL-c levels (17). Values of LDL-c were converted from mmol/L to mg/dL using a conversion factor of 38.67. The LDL/ApoB ratio was calculated from serum levels of LDL-c and ApoB (both in mg/dL). The LDL/ApoB ratio for estimating the prevalence of two LDL particle sizes was set as follows: LDL/ApoB < 1.2 (S-LDL) and LDL/ApoB > 1.2 (large dense [L-]LDL) (18). The ratio of TG to HDL-c (both in mmol/L) was logarithmically transformed and defined as the AIP (15). The AIP risk categories were classified as follows: AIP < 0.11 (low risk), AIP = 0.11-0.21 (moderate risk), and AIP > 0.21 (high risk).

### Statistical analysis

Statistical analysis was performed using the software StatsDirect Sales, version 2.7.9 (Cheshire M33, 3UY, UK). A graphical representation of the examined parameters was created using Excel 2019 (Microsoft Corporation, USA). The normality or non-normality of data distribution in each group was assessed using the Shapiro-Wilk’s *W* test. Categorical data were compared using the chi-square test or Fisher’s exact test, as appropriate. Differences in variables between groups were evaluated using parametric Student’s *t* test or nonparametric Mann-Whitney’s *U* test. The results are expressed as mean ± standard deviation for parametric variables or as median (25th percentile; 75th percentile) for nonparametric variables. *P* < 0.05 (two-sided) was considered statistically significant.

Patients with DM were divided into four groups according to the LDL/ApoB ratio. Differences in variables between these groups were analyzed using a one-way analysis of variance (ANOVA) test. *Post hoc* pairwise comparisons were performed at the Bonferroni-adjusted significance level of alpha for multiple comparisons (*p* = 0.0125 for four groups).

The relationships between the examined variables were evaluated using Spearman’s rank correlation test and are expressed by Spearman’s rank correlation coefficient rho (*r*) and *p* values. Fisher’s *r*-to-*z* transformation (*p* < 0.05) was used to evaluate the statistical significance of the difference between pairs of correlation coefficients within each set of comparisons.

## RESULTS

### SEX-RELATED BIOCHEMICAL PARAMETERS

The baseline characteristics of the patients with DM categorized by sex are listed in Table 1. Significantly lower HDL-c levels (*p* < 0.001) were observed in the DM-M group compared with the DM-F group. Levels of TG were similar in both sexes (*p* > 0.05). Values of AIP and LOOH were higher in the DM-M group than in the DM-F group, but this difference was not statistically significant (*p* > 0.05). The distribution of AIP risk categories in patients with DM showed a lower incidence of AIP < 0.11 (low-risk level) in men than in women (31% *vs.* 48%, respectively; *p* < 0.05) (Supplementary Table 1). In contrast, a higher incidence of AIP > 0.21 (high-risk level) was observed in men with DM than in women with DM (54% *vs.* 32%, respectively; *p* = 0.058). No significant differences between groups were found in the levels of other lipids or glycemic and oxidative stress parameters. Although a 67% prevalence of LDL/ApoB < 1.2 was observed in patients with DM, no difference between men and women was detected in this regard.

**Table 1:** Demographic and metabolic characteristics of patients with type 2 diabetes categorized by sex

Characteristics	DM-M group	DM-F group	p
N (%)	83 (64)	46 (36)	< 0.005
Age (years)	52.9 ± 6.1	52.4 ± 5.5	0.284
BMI (kg/m^2^)	31.5 (28.1; 33.6)	31.2 (27.9; 35.5)	0.348
Duration of diabetes (years)	3 (1; 6)	2 (1; 6)	0.456
History of HPT n (%)	49 (59)	26 (56)	0.116
History of CVD n (%)	16 (19)	2 (4)	< 0.05
Glucose-lowering therapy n (%)			
Insulin	9 (11)	2 (4)	0.106
Insulin + OHA	7 (8)	5 (11)	0.807
OHA	56 (68)	20 (43)	< 0.05
Diet	11 (13)	19 (41)	< 0.05
Lipid-lowering therapy	51 (61)	28 (61)	0.455
Fasting glycemia (mmol/L)	7.1 (6.2; 8.6)	6.9 (6.1; 7.5)	0.244
HbA1C (%)	6.8 (6.1; 7.5)	6.5 (6.1; 7.6)	0.216
(mmol/mol)	50.8 (43.1; 58.0)	47.0 (42.8; 59.0)	0.216
TC (mmol/L	4.6 (4.2; 5.4)	5.2 ± 1.2	0.056
HDL-c (mmol/L)	1.08 (0.98; 1.29)	1.31 ± 0.28	< 0.001
Non-HDL-c (mmol/L)	3.5 (2.9; 4.3)	3.9 ± 1.1	0.133
LDL-c (mmol/L)	2.6 (2.2; 3.2)	3.0 ± 0.9	0.078
TG (mmol/L)	1.8 (1.3; 2.4)	1.7 (1.2; 2.3)	0.368
ApoA1 (g/L)	1.62 ± 0.38	1.92 ± 0.24	0.068
ApoB (g/L)	0.85 ± 0.19	0.81 ± 0.36	0.066
LOOH (nmol/mL)	69.1 ± 25.4	62.3 ± 19.8	0.068
AIP	0.22 ± 0.27	0.13 ± 0.25	0.086
LDL/ApoB	1.11 ± 0.17	1.14 ± 0.12	0.258

Data are expressed as mean ± standard deviation for parametric variables or median (25th percentile; 75th percentile) for nonparametric variables. N (%) represents the number of subjects per group (percentage). P values were calculated using Student’s t test for normally distributed variables and Mann-Whitney’s U test for non-normally distributed variables (p < 0.05 was defined as statistically significant). Abbreviations: AIP, atherogenic index of plasma; ApoA1, apolipoprotein A1; ApoB, apolipoprotein B; BMI, body mass index; CVD, cardiovascular disease; DM-F, women with type 2 diabetes; DM-M, men with type 2 diabetes; HbA1c, glycated hemoglobin; HDL-c, high-density lipoprotein cholesterol; HPT, hypertension; OHA, oral hypoglycemic agents; LDL/ApoB, low-density lipoprotein cholesterol to apolipoprotein B ratio; LOOH, lipid hydroperoxides; TC, total cholesterol; TG, triglycerides.

In the control group, we observed a higher prevalence of AIP < 0.11 in women (91%, *p* = 0.025) than in men (data not shown). We found no difference in rates of LDL/ApoB < 1.2 between men and women in the control group (Supplementary Table 2).

### Biochemical parameters related to the LDL/ApoB ratio

Patients with DM were regrouped according to LDL/ApoB ratio as follows: LDL/ApoB < 1.2 (S-LDL group), n = 87 (56 men [S-LDL-M] and 31 women [S-LDL-F]) and LDL/ApoB > 1.2 (L-LDL group), n = 42 (27 men [L-LDL-M] and 15 women [L-LDL-F]). Although this distribution showed a higher incidence of LDL/ApoB < 1.2 than LDL/ApoB > 1.2 in both sexes (*p* < 0.05), the prevalence of LDL/ApoB < 1.2 in men was similar to that in women (67% and 66%, respectively). Significant differences in the values of HDL-c, TG, LOOH, and AIP were found between groups with/without LDL/ApoB < 1.2 in men and women (Table 2). Lower HDL-c levels (*p* < 0.005) and higher LOOH and AIP levels (both *p* < 0.05) were observed in the S-LDL-M group compared with the S-LDL-F group.

**Table 2:** Biochemical parameters in patients with type 2 diabetes categorized by sex and according to the low-density lipoprotein cholesterol to apolipoprotein B (LDL/ApoB) ratio

Characteristics	DM-Male group		DM-Female group		pa	p^b^	p^c^	p^d^
	S-LDL-M	L-LDL-M	S-LDL-F	L-LDL-F				
N (%)	56 (68)	27 (32)	31 (67)	15 (33)				
LDL/ApoB	1.02 ± 0.14	1.28 ± 0.06	1.08 ± 0.11	1.25 ± 0.04	< 0.0001	< 0.0001	0.064	0.171
Fasting glycemia (mmol/L)	7.8 ± 2.5	7.3 ± 2.4	7.5 ± 1.7	6.8 ± 1.5	0.224	0.238	0.358	0.514
HbA1C (%)	7.1 ± 1.2	6.7 ± 1.0	7.1 ± 1.3	6.8 ± 1.4	0.102	0.560	0.906	0.607
(mmol/mol)	54.0 ± 12.7	49.3 ± 11.0	54.2 ± 13.8	51.4 ± 14.9	0.102	0.560	0.906	0.607
TC (mmol/L)	4.6 ± 0.9	5.3 ± 1.3	5.2 ± 1.2	5.3 ± 1.1	< 0.005	0.668	< 0.05	0.874
HDL-c (mmol/L)	1.08 ± 0.22	1.25 ± 0.34	1.24 ± 0.26	1.43 ± 0.28	< 0.01	< 0.05	< 0.005	0.095
Non-HDL-c (mmol/L)	3.6 ± 0.8	4.0 ± 1.1	3.9 ± 1.2	3.9 ± 1.1	< 0.05	0.959	0.091	0.748
LDL-c (mmol/L)	2.5 ± 0.6	3.5 ± 0.9	2.9 ± 0.8	3.3 ± 0.9	< 0.0001	0.149	< 0.01	0.643
TG (mmol/L)	2.4 ± 1.2	1.2 ± 0.5	2.3 ± 1.0	1.3 ± 0.4	< 0.0001	< 0.005	0.672	0.664
ApoA1 (g/L)	1.66 ± 0.36	1.60 ± 0.49	1.93 ± 0.31	1.88 ± 0.29	0.782	0.407	0.181	0.087
ApoB (g/L)	0.84 ± 0.16	0.79 (0.71; 1.01)	0.80 ± 0.44	0.65 ± 0.23	0.801	0.076	0.093	0.488
ALOOH (nmol/mL)	0.84 ± 0.16	56.9 ± 13.7	65.1 ± 19.7	56.8 ± 19.5	< 0.005	< 0.05	< 0.05	0.994
AIP	0.32 ± 0.20	0.04 ± 0.23	0.23 ± 0.22	0.07 ± 0.17	< 0.0001	< 0.0001	< 0.05	0.695

Categorization of the prevalence of LDL/ApoB ratio by sex: S-LDL-M group, men with LDL/ApoB < 1.2 (small dense LDL); S-LDL-F group, women with LDL/ApoB < 1.2 (small dense LDL); L-LDL-M group, men with LDL/ApoB > 1.2 (large dense LDL); L-LDL-F group, women with LDL/ApoB > 1.2 (large dense LDL). N represents the number of subjects per subgroup. Data are expressed as mean ± standard deviation for parametric variables or median (25th percentile; 75th percentile) for nonparametric variables. P value indicates the statistical significance of the difference between the levels of biochemical parameters using a one-way analysis of variance (ANOVA) test and post hoc pairwise comparisons at p = 0.0125 (Bonferroni correction). ^a^p value between the S-LDL-M and L-LDL-M groups; ^b^p value between the S-LDL-F and L-LDL-F groups; ^c^p value between the S-LDL-M and S-LDL-F groups; ^d^p value between the L-LDL-M and L-LDL-F groups. Abbreviations: AIP, atherogenic index of plasma; ApoA1, apolipoprotein A1; ApoB, apolipoprotein B; HDL-c, high-density lipoprotein cholesterol; LDL-c, low-density lipoprotein cholesterol; LDL/ApoB, low-density lipoprotein cholesterol to apolipoprotein B ratio; LOOH, lipid hydroperoxides; TC, total cholesterol; HDL-c, high-density lipoprotein cholesterol; TG, triglycerides.

On the contrary, there were higher levels of TC (*p* < 0.05) and LDL-c (*p* < 0.01) in the S-LDL-F group than in the S-LDL-M group. Levels of AIP > 0.21 were found in both men and women with LDL/ApoB < 1.2. This indicates that patients of both sexes included in the S-LDL group represent a high-risk population. Significant differences in LOOH levels were observed between men and women in the groups with/without LDL/ApoB < 1.2. Levels of LOOH were higher in men and women with LDL/ApoB < 1.2 (*p* < 0.005 and *p* < 0.05, respectively) compared with men and women with LDL/ApoB > 1.2.

### Correlation analysis

**Figure 1:**
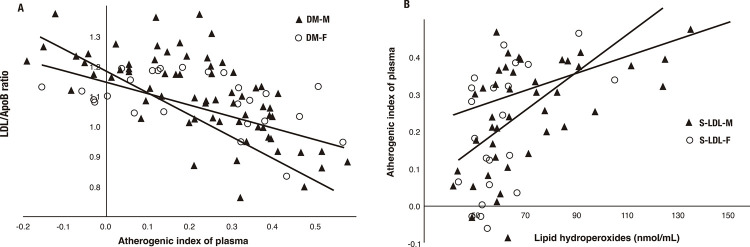
Correlations by sex between (A) low-density lipoprotein to apolipoprotein B ratio (LDL/ApoB) and atherogenic index of plasma (AIP) values in patients with type 2 diabetes (whole group) and between (B) AIP and lipid hydroperoxides (LOOH) values in patients with type 2 diabetes with LDL/ApoB < 1.2. (A) DM-M group (r = - 0.829, p < 0.0001) and DM-F group (r = - 0.670, p < 0.0001); p = 0.028 represents the statistical significance of the difference between two correlation coefficients (Fisher’s r-to-z transformation). (B) S-LDL-M group (r = 0.431, p < 0.001) and S-LDL-F group (r = 0.699, p < 0.005); p < 0.05 represents the statistical significance of the difference between two correlation coefficients (Fisher’s r-to-z transformation). Distribution of LDL/ApoB < 1.2 according to sex, in which the S-LDL-F and S-LDL-M groups correspond to women and men, respectively, with LDL/ApoB < 1.2 (small dense LDL).

In the first step, correlation analysis (Fisher’s *r*-to-*z* transformation) indicated stronger relationships of LDL/ApoB ratio with values of AIP (Figure 1A), TG, and LOOH in men with DM than in women with DM (Supplementary Table 3). However, stronger correlations of non-HDL-c with AIP and TG were found in the DM-F group (*p* < 0.05). Subsequently, the statistical significance of the difference between the pairs of correlation coefficients showed stronger correlations between AIP and LOOH (Figure 1B) and between TG and LOOH as well as ApoB in the S-LDL-F group than in the S-LDL-M group (Table 3). The same type of analysis did not show correlations between lipid risk parameters in the group of women with LDL/ApoB > 1.2 (data not shown).

**Table 3:** Results of Spearman's correlations between biochemical parameters and low-density lipoprotein cholesterol to apolipoprotein B (LDL/ApoB) ratio < 1.2 in patients with diabetes mellitus categorized by sex

Variables	S-LDL-M		S-LDL-F	
	r	p	r	p
Atherogenic index of plasma				
LOOH	0.431	< 0.001	* 0.699	< 0.005
Non-HDL-c	0.547	< 0.0001	0.686	< 0.0001
ApoB	0.542	< 0.0001	0.700	< 0.0001
TC	0.363	< 0.01	0.568	< 0.005
Triglycerides				
LOOH	0.416	< 0.001	* 0.669	< 0.005
Non-HDL-c	0.691	< 0.0001	0.820	< 0.0001
ApoB	0.692	< 0.0001	* 0.838	< 0.0001
TC	0.600	< 0.0001	0.776	< 0.0001

Categorization of the prevalence of LDL/ApoB < 1.2 by sex: S-LDL-F group, women with LDL/ApoB < 1.2 (small dense LDL); S-LDL-M group, men with LDL/ApoB < 1.2 (small dense LDL). P value represents the statistical significance of the differences between the analyzed variables in each group; r is Spearman's rank correlation coefficient (rho); * indicates the statistical significance of the difference between pairs of correlation coefficients (Fisher’s r-to-z transformation; p < 0.05). Abbreviations: ApoB, apolipoprotein B; LOOH, lipid hydroperoxides; non-HDL-c, non-high-density lipoprotein cholesterol; TC, total cholesterol.

## DISCUSSION

This study evaluated the correlations between atherosclerosis-related lipid parameters and LDL/ApoB < 1.2 in male *vs.* female patients with DM. Significantly lower HDL-c levels were observed in men with DM than in women with DM. Levels of TG were very similar between sexes, but AIP values were higher in men than in women, although this difference was not statistically significant. This indicates that lower HDL-c levels tend to elevate AIP values slightly, thereby increasing plasma atherogenicity in men with DM. In addition, the distribution of AIP risk categories according to sex among patients with DM revealed a higher incidence of AIP levels > 0.21 (corresponding to a high cardiovascular risk) in men compared with women. The AIP, reflecting the actual status of TG and HDL-c metabolism, may be involved in the pathophysiology of LDL particle size. Statistical analysis of the difference between correlation coefficients demonstrated stronger correlations between LDL/ApoB ratio and values of LOOH, AIP (Figure 1A), and TG in men with DM than in women with DM. However, these findings could be due to the categorization of patients with DM into two subgroups according to sex. Each subgroup (men with DM or women with DM) included individuals with LDL/ApoB both below and above 1.2.

Some reports have suggested that AIP and the LDL/ApoB ratio are relevant indicators of plasma atherogenicity and S-LDL particle prevalence and are considered independent predictors of cardiovascular risk (15,18,19,25). The causal role of LDL-c and other ApoB-containing lipoproteins, as well as strongly atherogenic S-LDL particles in the early stages of subclinical atherosclerosis, has been emphasized often (21,26). In our study, we calculated the AIP and LDL/ApoB ratio using laboratory-measured lipid parameters. Besides that, we considered this process of estimation of plasma atherogenicity and S-LDL particle prevalence to be adequate in terms of the spectrum of lipid parameters, which were measured in our study. Prevalent S-LDL particles in blood can be associated with increased oxidative modification of lipids (reflected in the increased LOOH level), which is involved in atherogenesis. Therefore, combined cardiovascular risk stratification using AIP (corresponding to three levels of risk categories) and LDL/ApoB ratio (corresponding to two LDL particle sizes) may be beneficial in clinical practice.

We recorded surprising results after dividing patients with DM according to sex and LDL/ApoB ratio. The prevalence of LDL/ApoB < 1.2 was significantly (*p* < 0.05) higher than that of LDL/ApoB > 1.2 in both men (68%) and women (67%). No differences in the prevalence of this ratio were detected between men and women. Higher HDL-c levels do not appear to rule out the occurrence of LDL/ApoB < 1.2. Men with LDL/ApoB < 1.2 had significantly lower levels of TC, HDL-c, and LDL-c and significantly higher values of AIP and LOOH than women in the S-LDL-F group. These findings indicate that significantly higher values of LOOH and AIP could correspond to increased lipid oxidative damage and plasma atherogenicity in men assigned to the S-LDL-M group. In contrast, higher levels of HDL-c and TC, and higher cutoff levels of LDL-c were found in the S-LDL-F group compared with the S-LDL-M group.

In our study, the strength of the relationships between lipid parameters changed significantly after the distribution of men and women according to LDL/ApoB ratio. Correlation analysis showed stronger correlations between multiple lipid parameters in the S-LDL-F group (women) compared with the S-LDL-M group (men) (Table 3). Exclusively, stronger correlations between values of AIP and LOOH (Figure 1B) and between values of TG and LOOH as well as ApoB were confirmed in women with LDL/ApoB < 1.2. Hypothetically, as the LDL/ApoB ratio decreases (reflecting a tendency to S-LDL particle predominance) with increasing values of AIP and LOOH (tendency to increase plasma atherogenicity and oxidative damage of lipids), cardiovascular risk will tend to elevate. These correlations indicate that women in the S-LDL-F group are at a potentially higher risk of future CVD than men with LDL/ApoB < 1.2. Moreover, correlation analysis did not confirm such correlation between lipid parameters in women with LDL/ApoB > 1.2 (data not shown).

We speculated whether these relationships between relevant lipid parameters due to LDL/ApoB < 1.2 in women (indicating a higher cardiovascular risk) could be linked to slightly higher levels of TC and higher cutoff levels of LDL-c and other factors, such as sex hormone levels. Recent studies have suggested that the prevalence of CVD during the menopausal period is increasing and, therefore, dyslipidemia as a key risk factor must be controlled in women with DM (27-29). We believe that further clinical studies are needed to investigate the mechanisms responsible for the effect of different sex hormone levels on lipid metabolism.

This study has some limitations related to the relatively small subgroups of men and women with LDL/ApoB < 1.2. Associations of the LDL/ApoB ratio with other lipid parameters between subgroups with/without a history of CVD in both sexes were not evaluated due to the low incidence of CVD among patients with DM. The findings of our study are consistent with reported opinions calling for future sex-related research focused on a larger sample of subjects with DM and including a higher proportion of women (2,5). Nevertheless, our study has some strengths represented by the study design with well-defined participant inclusion criteria and a wide range of lipid-related parameters not routinely used in clinical practice.

It is necessary to improve the prediction of CVD risk using surrogate lipid-related markers that could complement the commonly used biomarkers in clinical practice. Such a marker could be represented by the numerical value of the LDL/ApoB ratio reflecting the LDL particle size, the level of plasma atherogenicity, and atherosclerotic risk. This ratio can identify individuals with LDL/ApoB < 1.2 (with prevalent S-LDL particles and potentially increased cardiovascular risk) and distinguish them from those with prevalent L-LDL particles. Therefore, the LDL/ApoB ratio can significantly increase the predictive value of routinely used cardiovascular markers. Because laboratory methods for determining the size of LDL particles are difficult to apply in routine clinical practice due to their complex procedures and high cost, estimating the prevalence of S-LDL particles using the LDL/ApoB ratio may be beneficial for the early prevention of CVD in the general population.

In conclusion, identifying high-risk individuals using the LDL/ApoB ratio may have a supportive role in DM management, especially in menopausal women with specific thresholds of LDL-c and HDL-c levels. Due to its simplicity, the LDL/ApoB ratio has a promising future for screening and diagnostic purposes in clinical practice.

Sponsorship: this work was supported by the Ministry of Education, Science, Research and Sport of the Slovak Republic, Bratislava, Slovakia (VEGA grant numbers: 1/0451/16 and 1/0200/22).

## Appendix

**Supplementary Table 1:** Sex-related distribution of atherogenic index of plasma (AIP) levels in patients with type 2 diabetes

AIP risk categories	DM-M n = 83	DM-F n = 46	p
AIP < 0.11 n (%)	26 (31)	22 (48)	< 0.05
AIP = 0.11 – 0.21 n (%)	12 (15)	9 (20)	0.371
AIP > 0.21 n (%)	45 (54)	15 (32)	0.058

Risk categories by AIP: AIP < 0.11 (low risk), AIP = 0.11 – 0.21 (moderate risk), AIP > 0.21 (high risk). N (%) is the number of subjects per group (percentage); p values represent the statistical significance of the difference between groups. Abbreviations: AIP, atherogenic index of plasma (log[TG/HDL]); DM-F, women with type 2 diabetes; DM-M, men with type 2 diabetes.

**Supplementary Table 2:** Demographic and metabolic characteristics of the participants in the control group categorized by sex

Characteristics	CG-M group	CG-F group	p
N (%)	38 (62)	23 (38)	< 0.05
Age (years)	51.3 ± 7.2	50.7 ± 6.5	0.835
BMI (kg/m^2^)	27.1 ± 3.2	25.3 ± 5.4	0.194
Fasting glycemia (mmol/L)	5.1 ± 0.4	4.7 ± 0.5	0.156
TC (mmol/L)	5.3 ± 0.9	5.3 ± 0.7	0.781
HDL-c (mmol/L)	1.35 ± 0.29	1.59 ± 0.42	< 0.05
Non-HDL-c (mmol/L)	3.9 ± 0.9	3.7 ± 0.6	0.396
LDL-c (mmol/L)	3.3 ± 0.8	3.3 ± 0.5	0.770
TG (mmol/L)	1.1 (0.9; 1.6)	0.9 (0.7; 1.1)	< 0.05
ApoA1 (g/L)	1.84 ± 0.41	2.11 ± 0.51	< 0.05
ApoB (g/L)	0.94 ± 0.19	0.87 ± 0.16	0.167
LOOH (nmol/mL)	52.1 (48.0; 59.5)	52.2 (44.1; 57.3)	0.312
AIP	-0.05 ± 0.12	-0.22 ± 0.13	< 0.005
LDL/ApoB	1.39 ± 0.16	1.44 ± 0.18	0.268

Data are expressed as mean ± standard deviation for parametric variables or median (25th percentile; 75th percentile) for nonparametric variables. N (%) represents the number of subjects per group (percentage); p values were calculated using Student’s t test for normally distributed variables and Mann-Whitney’s U test for non-normally distributed variables (p < 0.05 was defined as statistically significant). Abbreviations: ApoA1, apolipoprotein A1; ApoB, apolipoprotein B; AIP, atherogenic index of plasma (log[TG/HDL]); BMI, body mass index; CG-F; women in the control group; CG-M; men in the control group; HDL-c, high-density lipoprotein cholesterol; LDL-c, low-density lipoprotein cholesterol; LOOH, lipid hydroperoxides; TC, total cholesterol; TG, triglycerides.

**Supplementary Table 3:** Results of Spearman's correlations between biochemical parameters in patients with diabetes mellitus categorized by sex

Variables	DM-M group		DM-F group	
	r	p	r	p
LDL/ApoB ratio				
TG	*-0.843	< 0.0001	-0.691	< 0.0001
HDL-c	0.291	< 0.01	0.322	< 0.05
AIP	*-0.829	< 0.0001	-0.670	< 0.0001
LOOH	*-0.598	< 0.0001	-0.347	0.040
Atherogenic index of plasma				
Non-HDL-c	0.307	0.002	* 0.572	< 0.0001
LOOH	0.571	< 0.0001	0.460	< 0.005
Triglycerides				
Non-HDL-c	0.366	<0.001	* 0.681	<0.0001
HDL-c	-0.263	0.008	-0.295	0.031
LOOH	0.556	< 0.0001	0.539	< 0.005

P value represents the statistical significance of the difference between analyzed variables in study groups; r is Spearman's rank correlation coefficient (rho); * indicates the statistical significance of the difference between pairs of correlation coefficients (Fisher’s r-to-z transformation; p < 0.05). Abbreviations: AIP, atherogenic index of plasma; DM-F, women with type 2 diabetes; DM-M, men with type 2 diabetes; HDL-c, high-density lipoprotein cholesterol; LOOH, lipid hydroperoxides; TG, triglycerides.

**Supplementary Table 4:** Biochemical parameters in patients with type 2 diabetes categorized by sex and lipid-lowering

Characteristics	DM-Male group		DM-Female group		pa	p^b^	p^c^	p^d^
	Statin-M	Without-M	Statin-F	Without-F				
N (%)	51 (61)	32 (39)	28 (61)	18 (39)				
Fasting glycemia (mmol/L)	7.5 ± 2.4	8.1 ± 2.5	7.2 ± 1.3	7.3 ± 2.3	0.255	0.714	0.518	0.334
HbA1_C_ (%)	6.9 ± 1.1	7.0 ± 1.1	7.1 ± 1.4	6.8 ± 1.1	0.678	0.462	0.464	0.560
(mmol/mol)	52.0 ± 12.4	53.2 ± 12.3	54.3 ± 15.2	50.8 ± 11.7	0.678	0.462	0.464	0.560
TC (mmol/L)	5.0 ± 1.1	4.6 ± 0.9	5.1 ± 1.2	5.4 ± 0.9	0.073	0.340	0.689	< 0.005
HDL-c (mmol/L)	1.18 ± 0.30	1.07 ± 0.21	1.31 ± 0.31	1.29 ± 0.24	0.094	0.861	0.064	< 0.005
Non-HDL-c (mmol/L)	3.8 ± 0.9	3.5 ± 0.8	3.8 ± 1.2	4.1 ± 0.9	0.162	0.314	0.908	< 0.05
LDL-c (mmol/L)	2.9 ± 0.9	2.7 ± 0.8	2.9 ± 1.0	3.2 ± 0.6	0.384	0.147	0.862	< 0.05
TG (mmol/L)	2.1 ± 1.3	1.8 ± 0.9	2.0 ± 1.1	1.8 ± 0.7	0.295	0.796	0.616	0.874
ApoA1 (g/L)	1.69 ± 0.38	1.53 ± 0.40	1.93 ± 0.31	1.85 ± 0.36	0.343	0.623	0.239	0.452
ApoB (g/L)	0.86 ± 0.12	0.75 ± 0.26	0.64 ± 0.29	1.27 ± 0.31	0.167	< 0.05	< 0.05	< 0.05
LOOH (nmol/mL)	71.3 ± 24.2	65.8 ± 27.2	60.3 ± 15.9	66.5 ± 26.9	0.344	0.378	< 0.05	0.965
AIP	0.24 (0.05; 0.35)	0.23 (0.08; 0.37	0.06 (-0.08; 0.33)	0.11 (0.07; 0.28)	0.817	0.890	0.250	0.479
LDL/ApoB	1.10 ± 0.17	1.12 ± 0.16	1.12 ± 0.14	1.17 ± 0.08	0.662	0.191	0.571	0.214

Categorization of lipid-lowering therapy by sex: Statin-M group, men receiving statin therapy; Without-M group, men receiving no therapy; Statin-F group, women receiving statin therpy; Without-F group, women not receiving therapy. N represents the number of subjects per subgroup. Data are expressed as mean ± standard deviation for parametric variables or median (25th percentile; 75th percentile) for nonparametric variables. P value indicates the statistical significance of the difference between the levels of biochemical parameters using a one-way analysis of variance (ANOVA) test and post hoc pairwise comparisons at p = 0.0125 (Bonferroni correction). ap value between the Statin-M and Without-M groups; ^b^p value between the Statin-F and Without-F groups; ^c^p value between the Statin-M and Statin-F groups; ^d^p value between the Without-M and Without-F groups. Abbreviations: AIP, atherogenic index of plasma; ApoA1, apolipoprotein A1; ApoB, apolipoprotein B; HbA1c, glycated hemoglobin; HDL-c, high-density lipoprotein cholesterol; LDL/ApoB, low-density lipoprotein cholesterol to apolipoprotein B ratio; LOOH, lipid hydroperoxides; TC, total cholesterol; TG, triglycerides.
